# Addressing the worldwide shortages of face masks

**DOI:** 10.1186/s42833-020-00015-w

**Published:** 2020-07-31

**Authors:** Dongxiao Ji, Li Fan, Xiaoxia Li, Seeram Ramakrishna

**Affiliations:** 1 Department of Mechanical Engineering, National University of Singapore, Singapore, 117574 Singapore; 2 Department of Materials Science and Engineering, College of Engineering, Peking University, Beijing, 100871 China

**Keywords:** COVID-19, Epidemic prevention measures, Mask, Reusable, Antiviral, Sustainable development, Transmission of virus

## Abstract

The year 2020 will be punctuated by coronavirus disease 2019 (COVID-19) in the history of human civilization. Within four months, COVID-19 has become a public healthcare crisis in all nations around the world. Until a suitable vaccine is found and made widely available, the immediate solutions to protect individuals and to control the spread of the pandemic include wearing a face mask, maintaining personal hygiene, and social distancing. Certified face masks have become national essentials, and countries have imposed restrictions on exports, which has increased the worldwide shortages of masks and raw materials. This situation has also led to confusion and misinformation about face masks. This paper aims to provide quality information on face masks to alleviate the shortages. Disinfecting used masks and making homemade masks are discussed as emergency solutions. The development and manufacture of innovative masks (such as reusable masks, antivirus masks, and degradable masks) have become essential needs of society and involve both opportunities and challenges during this unprecedented time. In this prospective study, we provide the definitions, basic requirements, materials, possible preparation methods, and challenges of these innovative masks and highlight their important role in preventing epidemics similar to COVID-19.

## Background

The coronavirus disease 2019 (COVID-19) pandemic has caused major changes in nearly every aspect of life in the first 6 months of 2020 [1]. The number of confirmed cases is close to 6.5 million, and new cases increase by ~ 100,000 per day (as of June 5, 2020) [2]. Until a suitable vaccine is developed and made widely available, wearing a protective facemask, personal hygiene and social distancing are ubiquitously followed to prevent the spread of the virus. Certified face masks have become national essentials. The COVID-19 outbreak in China, which produces half of the world’s face masks, has generated a twofold challenge: there is surging domestic demand and a major disruption to the global supply. It is estimated that 89 million medical masks will be required every month and a 40% increase in manufacturing is expected to meet the global demand [3]. Because masks protect people from viral infection, there is a worldwide surge in the use of billions of face masks every day. This situation will continue and has accentuated the worldwide shortages of masks. The situation also led to confusion and misinformation about face masks. Here, we discuss issues such as (A) the transmission of the virus, (B) different types of masks and their intended purposes, (C) the effectiveness of masks in controlling the pandemic, (D) different designs of masks and their usefulness, (E) different manufacturing methods and production strategies, (F) the effectiveness of disinfection or sterilization methods for reusing masks, (G) anti-viral masks, (H) environmentally friendly degradable masks, (I) materials selection and strategies, and (J) standards and methods for testing and evaluation of the performance of masks. Overall, the purpose of this manuscript is to provide facts about face masks to alleviate shortages.

## Main text

### The transmission of SARS-CoV-2

**Fig. 1:**
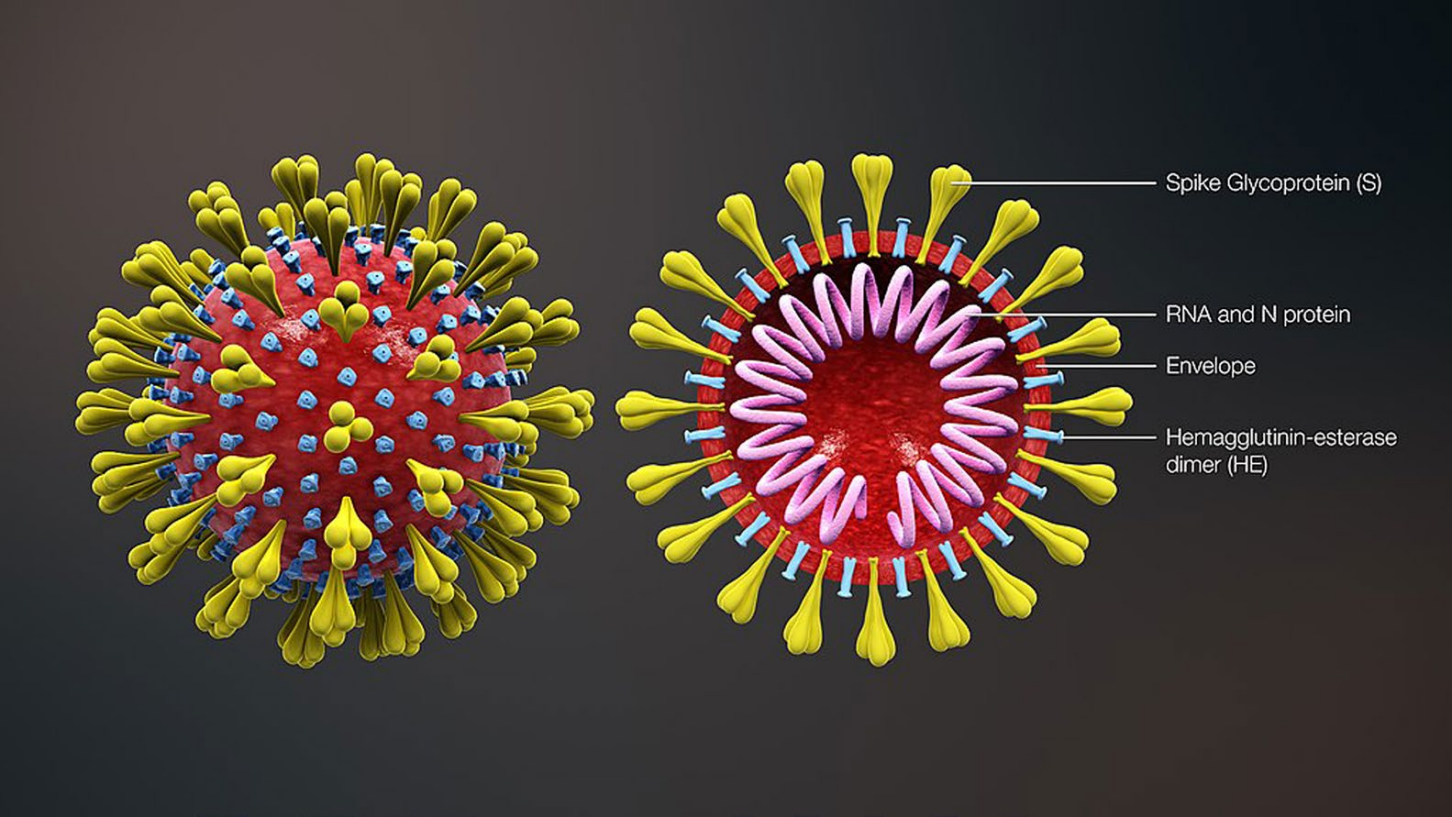
Structure of SARS-CoV-2. Reproduced with permission from [4]. Copyright 2020, https://creativecommons.org/licenses/by-sa/4.0

The virus called severe acute respiratory syndrome coronavirus 2 (SARS-CoV-2) is the main cause of the disease outbreak (Fig. 1). Similar to SARS-CoV (a virus outbreak in 2003), SARS-CoV-2 can combine with angiotensin-converting enzyme 2 (ACE2) to invade the human body. The infected patient exhibits flu-like symptoms accompanied by increased body temperature or cough. Severely affected patients may develop pneumonia or acute respiratory distress syndrome (ARDS). Because SARS-CoV-2 can make better use of ACE2, it can invade the human body more easily than SARS-CoV [5]. Statistics indicate that the R0 value (i.e., the average number of other patients to whom an infectious disease is transmitted from one patient) of SARS-CoV-2 is almost double that of SARS-CoV (5.7 vs 3.2) [6, 7]. More importantly, since the virus has a long incubation period (3–20 days) and given the presence of asymptomatic carriers, preventing transmission of SARS-CoV-2 is challenging.

**Fig. 2:**
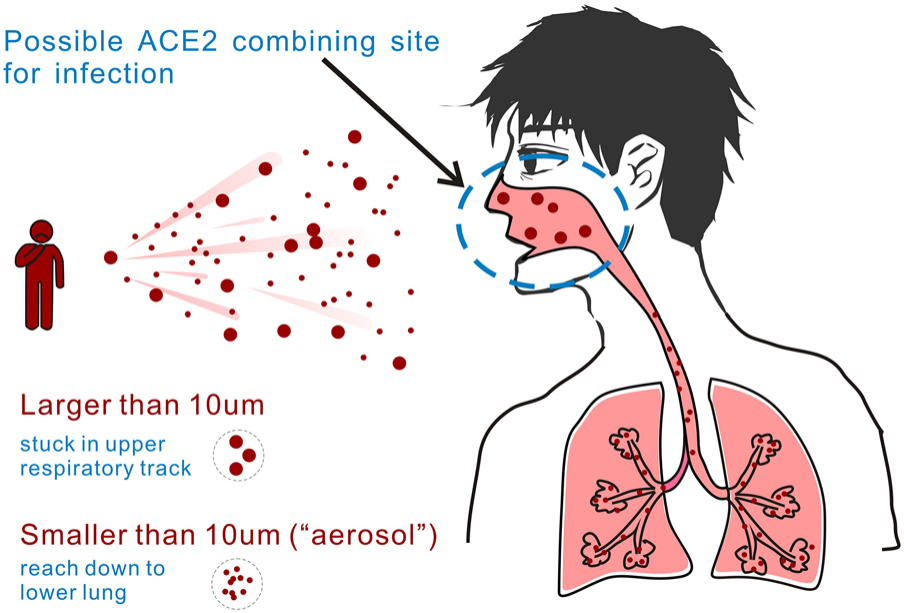
Transmission mode of SARS-CoV-2 from human-to-human

Generally, ACE2 expression in the lung is only a few molecules per alveolar cell. Surprisingly, a recently published paper reported that ACE2 is highly expressed in some types of cells of the inner nose [8]. Therefore, when the virus enters the nasal cavity, it can easily combine with ACE2 to invade human cells. Short-range aerosols and droplets are the main routes of SARS-CoV-2 transmission (Fig. 2) [9]. The virus is released into the air when an infected person breathes, coughs, sneezes, or even sings. Singing has been found to be comparable to continuous coughing in the transmission of airborne pathogens [10], as demonstrated by a choir practice on March 10, 2020, in Washington state, USA. Choir members stated that they did not touch each other during the practice; but 45 out of the 60 members were diagnosed with the SARS-CoV-2 virus within 3 weeks, and two of them unfortunately passed away.

### Classification of masks

Masks can be divided into four categories according to their filtering capacity (Table 1): (1) face-covering masks (for example, homemade cloth masks), (2) surgical masks, (3) medical protective masks (mainly used to prevent airborne particles, such as N95 and FFP masks), and (4) occupational protective masks (mainly used for occupational protection, such as particulate and gas respirators). To provide respiratory protection against particles or airborne, masks should meet a particulate filtration efficiency of 80% or more.

**Table 1:** Classification and characteristics of masks

Types	Face covering masks	Surgical masks	N95 mask	Surgical respirators
Purpose	Prevents large particles (> 10 um) expelled by the wearer from reaching the environment	Prevents large particles (> 10 um) expelled by the wearer from reaching the environment To be used as a physical barrier to protect people from large droplets of blood or body fluids	Reduces exposure to very small airborne particles or contaminants May not protect against sprays and direct liquid splashes	Provides the protection of both a surgical mask and N95 respirator To be used as a physical barrier for large droplets of blood or body fluids as well as very small particles (e.g., fine aerosolized droplets), such as those produced by coughing
Fit	Does not fit tightly	Does not fit tightly	Tight fit	Tight fit
Filtration efficiency	Windproof, keep warm, isolate large particles such as dust	Bacterial filtration efficiency above 95%	Minimum 95% against particulate aerosols (of 0.3 micron in size) free of oil	Minimum 95% against particulate aerosols (of 0.3 micron in size) free of oil
Fluid resistance (i.e., resistance to penetration of bodily fluids)	Not fluid resistant	Yes	Not tested for fluid resistance	Tested to be fluid resistant

### “Masking strategy” to decrease community transmission of the virus

**Fig. 3:**
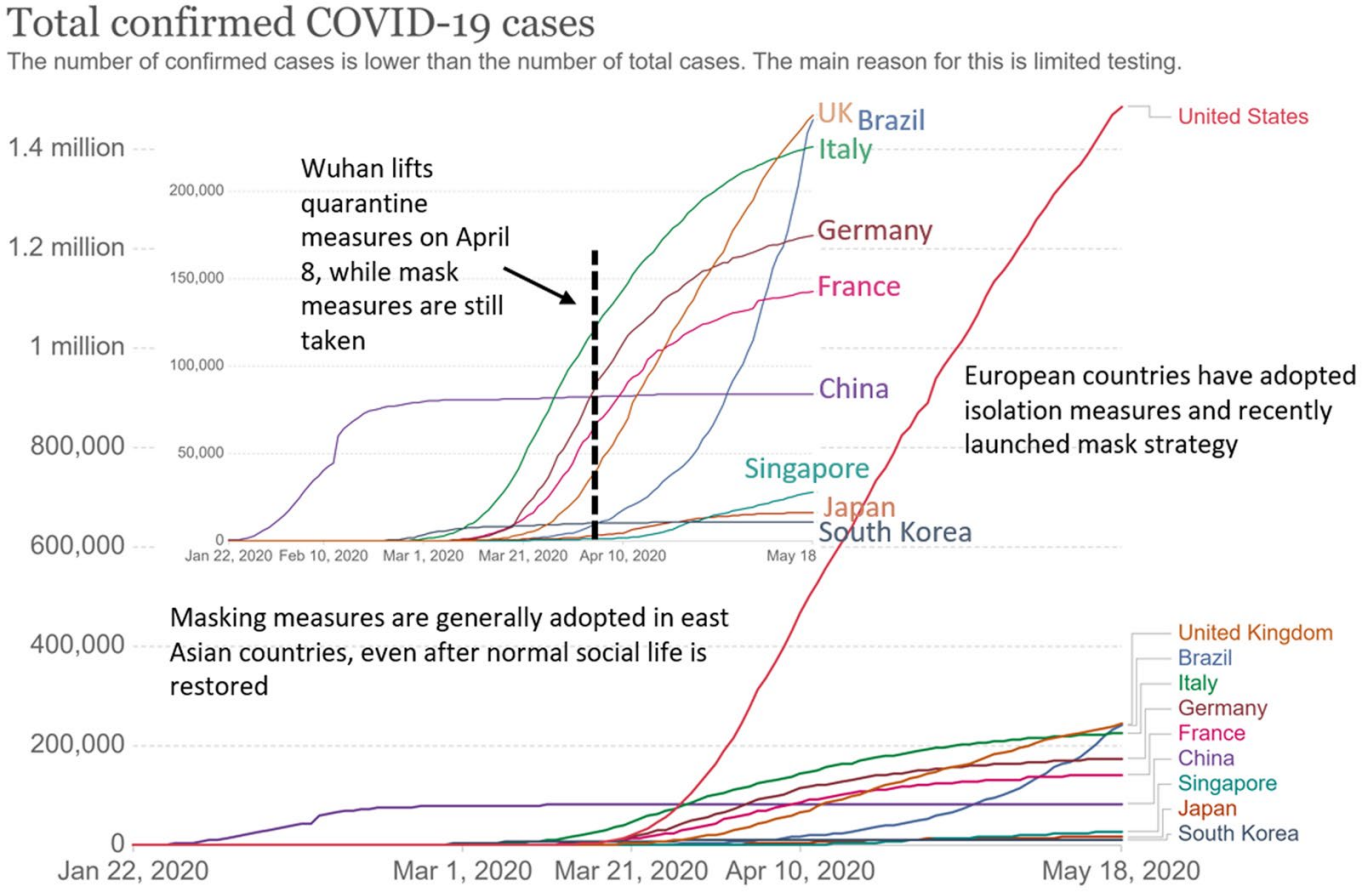
As of May 18, 2020, the confirmed cases of COVID-19 in several countries. Data collected from WHO Coronavirus disease 2019 (COVID-19) situation reports [11]

At the beginning of the outbreak, each country adopted different epidemic prevention measures. The policies include school and workplace closures, cancellation of public events and gatherings, stay-at-home restrictions, and testing and contact tracing. Most European and American countries recommend that residents stay at home and follow up to isolate infected and potentially infected persons. The number of infected people in these countries continues to rise after approximately two months of measures. Some countries, such as Italy, Germany, France, Singapore, and the United Kingdom, have recently (as of the end of May 2020) begun to encourage residents to wear masks. The curve of these countries started to flatten (Fig. 3).

In contrast, due to cultural differences, many East Asian countries encouraged or even mandated that everyone wear masks from the beginning of the outbreak. These countries have flattened the curve, even at the initial stage (such as Japan and South Korea, Fig. 3).

Statistics show that after the lifting of the lockdown on April 8 in Wuhan, China (the city where the outbreak began), the number of infections has increased only slightly due to the continued implementation of the masking policy. Currently (May 2020), most cities in China have basically returned to normal economic order under the premise of wearing masks. Similar countries include Japan and South Korea.

In a study published on April 22, 2020, researchers proposed two artificial intelligence (AI) models designed to predict the impact of wearing masks on the spread of the new coronavirus [12]. One of the models attempts to predict the shielding effect of wearing a mask compared to other measures (mainly closures and physical distance). By changing the parameters that affect the degree of interaction and the average number of intimate contacts, researchers can use the model to measure various degrees of social alienation and blockade measures. The AI simulation showed that when the adoption rate reached 80%, the degree to which the infection curve flattened due to mask wearing was much greater than the degree to which it flattened due to closure (the former caused 60,000 deaths, while the latter caused 180,000, a three-fold difference). At the same time, the 50% mask penetration rate is not sufficient to prevent continued spread (which will cause 240,000 deaths). Since May 31, 2020, social isolation replaced strict closures; unless masks are worn, the spread will be uncontrollable.

The second model uses agent-based technology, and each agent “wears” different kinds of masks. These simulation results show that if a mask is worn early enough, even if the mask is nonmedical or homemade, it can reduce the spread of the virus. If the mask adoption rate reaches 100% during an outbreak, the number of infections will be “dramatically” reduced.

Both models indicate that if 80% of people start wearing cloth masks in public places before the lockdown is lifted, the spread of the outbreak will be limited enough to lift the lockdown and avoid a second wave of infections. If masking measures are not taken, once the lockdown is lifted, even if social isolation continues, the virus will still infect almost half of the population. If 100% of the population wears masks, even after 300 days, the curve will remain at basically a constant level, and the prevalence rate will be less than 10% when society is completely liberalized.

These simulation results are consistent with the actual data, at least so far (as of the end of May 2020). Therefore, governments and international agencies have suggested using masking strategies as one of the key tools in the epidemic prevention policy. Since this virus is expected to affect humans for some time, people may have to wear masks for a long time in the future.

### Strategies for alleviating shortages of masks

Statistics and theoretical simulations have confirmed that mask-wearing plays an important role in controlling the spread of the SARS-CoV-2 virus. However, the problem faced by many governments is that they are unable to provide enough masks for everyone, especially for frontline medical staff. Furthermore, the disposable masks currently used add a new source of pollution to society. This is not only a waste of resources but also further increases the burden on the ecological environment.

**Fig. 4:**
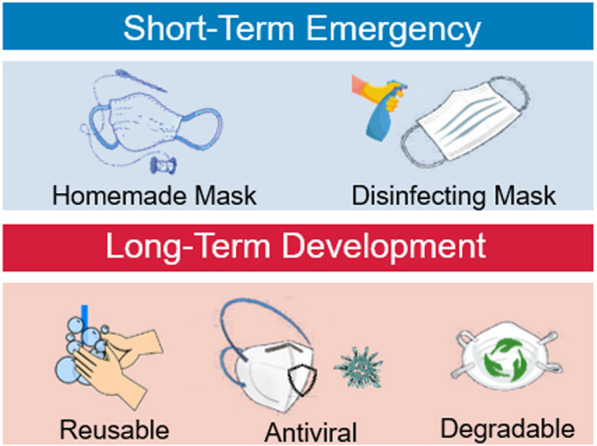
Strategies for alleviating shortages of masks

There are several strategies that have the potential to solve the problem of the shortage of masks (Fig. 4). Disinfecting used masks using appropriate methods and making homemade masks are two emergency solutions in times of mask shortage. In the long run, innovative masks, including reusable masks, antivirus masks, and degradable masks, will be of great significance for virus protection and environmental protection and to alleviate shortages of masks.

### Sterilization methods for reusing masks

For filtering facepiece respirators (FFRs), the sterilization strategies for the extended use and reuse are currently available from the National Institute for Occupational Safety and Health (NIOSH) [13]. The reduce of the pathogen burden, maintain the function of the FFRs, and present no residual chemical hazard are expected for an effective mask decontamination.

VHP, UVGI, and MH are the most promising disinfection strategy toward the used FFRs (method and performance have been listed in Table 2). It worth noting that many of these methods can only be used for limited times. No current data support the effectiveness of these decontamination methods for FFRs specifically against SARS-CoV-2. Therefore, even after decontamination, these FFRs should be handled carefully.

**Table 2:** Sterilization method and effect on mask performance

Method	Treatment level	Fitting performance	Filtration performance	Antimicrobial performance
Vaporous hydrogen peroxide (VHP)	Usually use commercial hydrogen peroxide steam generator. Directly use VHP to treat the surface of the mask, generally 10–30 min.	Passed (Fitting unaffected for up to 20 cycles)	Passed	Can be maintained (> 99.99%)
Ultraviolet germicidal irradiation (UVGI)	Ultraviolet germicidal irradiation to treat the surface of the mask. 0.5–950 J/cm^2^ were used	depending on model (90–100% can pass)	Passed	Can be maintained (99.9% for all tested viruses)
Microwave generated steam	microwave models: 1100–1250 W 40 s treatment for 2 min	95–100% Passed	Passed, even after 20 cycles	Can be maintained
Microwave steam bags	microwave models: 1100 W, 90 s treatment, 60 mL tap water was filled in the bags	Not evaluated	Passed	Can be maintained
Moist heat (MH) incubation	15–30 min treatment at 60 °C	Passed	Passed	Can be maintained
Liquid hydrogen peroxide	1 s to 30 min treatment with Liquid hydrogen peroxide concentration of 3–6%	Not evaluated	Passed	Not evaluated
Ethylene oxide	1 h at 55 °C; conc. range: 725–833 mg/L	Not evaluated	Passed	Not evaluated

Courtesy of the CDC website [13]

Liao et al. [14] also provided advice on sterilization methods and the durability of N95 respirators. Their experimental results show that the filtering performance of the N95 respirator can be maintained for approximately 50 cycles by heating (dry or in the presence of humidity) < 100 °C. The ultraviolet (UV) irradiation method is also useful, but it can only last for 20 cycles. Other methods, including soaking in ethanol and chlorine-based disinfection water for a short time, greatly reduce the filtration performance of N95 respirators.

For the commonly used commercial surgical mask, there are currently no corresponding experimental data that can support a reasonable method of disinfection and reuse. The filter layer of these masks is made of PP non-woven fabric with a diameter of 0.5–1 µm. This is the same as the filter layer of N95 and FFP masks except that the thickness of the filter layer in the surgical mask is thinner. According to the above disinfection experience for N95 and FFP masks, moist heat and ultraviolet irradiation may be an efficient optional disinfection method for the commonly used surgical mask. It is worth noting that this can only be used as an emergency measure when no mask is available. Because the quality of commercial masks and the sources of raw materials differ, the results may vary greatly when using a sterilization method for masks from different manufacturers. Governments or industry associations can encourage mask manufacturers to provide appropriate disinfection methods and performance data for their products by establishing relevant standards, which will have a positive effect on alleviating the shortage of masks.

### Homemade masks

The use of homemade masks is an emergency measure for epidemic protection under the premise of a shortage of masks. This type of mask is also called a cloth face covering since most of these masks are made of fabrics that are available in the home.

The CDC recommends wearing cloth face coverings in public settings where other social distancing measures are difficult to maintain (e.g., grocery stores and pharmacies), especially in areas of significant community-based transmission [15]. It also advises the use of simple cloth face coverings to slow the spread of the virus and help people who may have the virus and not know it from transmitting it to others. Cloth face coverings fashioned from household items or made at home from common materials at low cost can be used as an additional, voluntary public health measure.

Researchers from the University of Chicago conducted a timely study and showed that by reasonably selecting and matching fabrics, even homemade masks can achieve more than 95% filtration efficiency (Table 3).

**Table 3:** Comparison of the filtration performance of masks made of different materials [16]

Sample	Flow rate: 1.2 CFM		
	Filter efficiency (%)		Pressure differential
	< 300 nm average ± error	> 300 nm average ± error	ΔP (Pa)
N 95	85 ± 15	99.9 ± 0.1	2.2
Surgical mask	76 ± 22	99.6 ± 0.1	2.2
Cotton quilt	96 ± 2	96.1 ± 0.3	2.7
Quilter’s cotton (80TPI), 1 layer	9 ± 13	14 ± 1	2.2
Quilter’s cotton (80TPI), 2 layers	38 ± 11	49 ± 3	2.5
flannel	57 ± 8	44 ± 2	2.2
Cotton (600TPI), 1 layer	79 ± 23	98.4 ± 0.2	2.5
Cotton (600TPI), 2 layers	82 ± 19	99.5 ± 0.1	2.5
Chiffon, 1 layer	67 ± 16	73 ± 2	2.7
Chiffon, 2 layers	83 ± 9	90 ± 1	3.0
Natural silk, 1 layer	54 ± 8	56 ± 2	2.5
Natural silk, 2 layers	65 ± 10	65 ± 2	2.7
Natural silk, 4 layers	86 ± 5	88 ± 1	2.7
Cotton/chiffon	97 ± 2	99.2 ± 0.2	3.0
Cotton/silk	94 ± 2	98.5 ± 0.2	3.0
Cotton/flannel	95 ± 2	96 ± 1	3.0

TPI = Threads per inch

We provide suggestions for homemade masks with regard to materials: (i) the filtration performance of single-layer fabrics is usually poor, and the use of multiple layers (> 4 layers) can provide better protection for individuals; (ii) since synthetic fibers rubbing against each other may generate static electricity, the filtering performance of synthetic fiber fabrics (such as polyester, nylon, or acrylic) may be better than that of natural fiber fabrics (cotton or cellulose fiber); (iii) the use of cotton fabrics combined with synthetic fiber fabrics may maintain the uniformity of static electricity.

It is worth noting that current research on homemade masks is still insufficient. Many suggestions are based on experience, and there is not enough scientific basis for reference. Opportunities for future studies include examining how long the virus can survive on this type of mask, standard disinfection and cleaning procedures, and whether the wearing environment affects the performance of the covering mask. Until these problems are scientifically supported, homemade masks can only be an emergency measure to solve the shortage of masks.

### Reusable masks

Due to the shortages, demand and supply mismatch, and inaccessibility due to high prices in several countries, the reusability of medical grade masks has been considered to satisfy the growing demand. Many information and self-help videos on the internet explain how to make cloth-type reusable masks and use them properly [15]. Creative ideas and innovations on how to improve the filtration efficiency of cloth-type reusable masks are also available [16].

With regard to the reusability of standard surgical face masks, a good reusable mask against COVID-19 should provide high particulate filtration efficiency and bacterial filtration efficiency comparable to standard surgical masks. A good reusable mask should have durability. In other words, after proper disinfection or washing, its appearance, fit, filtration efficiency and breathing resistance should not change significantly. A quality reusable mask should meet the requirements of relevant standards (the key criteria are extracted from the standards and listed in Table 4) after being disinfected or washed using an appropriate method. Furthermore, it is expected that such masks can still meet the requirements of relevant standards after 50 (or more) proper sterilization treatments.

**Table 4:** Standards for facepiece respirators

Certification/class (standard)	N95(NIOSH-42CFR84)	FFP2 (EN 149-2001)	KN95 (GB2626-2006)	P2 (AS/NZ 1716:201)	Korea 1st Class (KMOEL - 2017-64)	DS (Japan JMHLW Notification 214, 2018)
Filter performance – (must be ≥ X % efficient)	≥ 95%	≥ 94%	≥ 95%	≥ 94%	≥ 94%	≥ 95%
Test agent	NaCl	NaCl and paraffin oil	NaCl	NaCl	NaCl and paraffin oil	NaCl
Flow rate	85 L/min	95 L/min	85 L/min	95 L/min	95 L/min	85 L/min
Total inward leakage (TIL)*– tested on human subjects each performing exercises	N/A	≤ 8% leakage (arithmetic mean)	≤ 8% leakage (arithmetic mean)	≤ 8% leakage (individual and arithmetic mean)	≤ 8% leakage (arithmetic mean)	Inward Leakage measured and included in User Instructions
Inhalation resistance – max pressure drop	≤ 343 Pa	≤ 70 Pa (at 30 L/min) ≤ 240 Pa (at 95 L/min) ≤ 500 Pa (clogging)	≤ 350 Pa	≤ 70 Pa (at 30 L/min) ≤ 240 Pa (at 95 L/min)	≤ 70 Pa (at 30 L/min) ≤ 240 Pa (at 95 L/min)	≤ 70 Pa (w/valve) ≤ 50 Pa (no valve)
Flow rate	85 L/min	Varied – see above	85 L/min	Varied – see above	Varied – see above	40 L/min
Exhalation resistance - max pressure drop	≤ 245 Pa	≤ 300 Pa	≤ 250 Pa	≤ 120 Pa	≤ 300 Pa	≤ 70 Pa (w/valve) ≤ 50 Pa (no valve)
Flow rate	85 L/min	160 L/min	85 L/min	85 L/min	160 L/min	40 L/min
Exhalation valve leakage requirement	Leak rate ≤ 30 mL/min	N/A	Depressurization to 0 Pa ≥ 20 s	Leak rate ≤ 30 mL/min	Visual inspection after 300 L/min for 30 s	Depressurization to 0 Pa ≥ 15 s
Force applied	− 245 Pa	N/A	− 1180 Pa	− 250 Pa	N/A	− 1470 Pa
CO_2_ clearance requirement	N/A	≤ 1%	≤ 1%	≤ 1%	≤ 1%	≤ 1%

In principle, the filtering efficiency of the mask is the decisive factor. The masks commonly used at present (including surgical masks, N95 and FFP masks) are filtered by the mechanism of static electricity (electrostatic electret treatment of melt-blown non-woven filter). This filtering method is very effective for particles with a diameter of less than 0.3 µm, and it can usually achieve more than 90% filtration efficiency. However, static electricity is easily lost, especially after washing or wearing for a long time; hence, this type of mask is meant to be disposable.

For the design of a reusable mask, there is a more effective filter called the “nanofiber filter” that does not rely on static electricity to filter dust and droplets. It uses a smaller pore size and good pore distribution to physically filter aerosol particulates comprising viruses or harmful dust [17]. After use, it can be disinfected in a suitable way. As long as the physical structure of the nanofiber filter is not damaged, it will maintain the original filtration performance. Using this filtration mechanism, Korean scientists have prepared reusable mask prototypes with nanofibers [18]. After undergoing 20 rounds of washing with an ethanol sterilizing solution, the nanofiber design was able to successfully filter 94% of contaminants (namely, bacteria with ethanol) and retained its original shape. Hence, such innovative masks have the potential for reusability and longer life.

During the outbreak, many countries experienced challenges in procuring sufficient quantities of quality masks. Properly designed and validated reusable masks can protect people during similar outbreaks, prevent the spread of viruses and save resources. At the same time, if everyone wears a mask, this can also help to alleviate the anxiety of the public. Reusable masks will play an important role in pandemic spread prevention and control in special circumstances.

### Antivirus masks

An anti-virus mask is a mask that kills or eliminates viruses in a short time while ensuring basic filtration performance while wearing.

Anti-virus masks are an essential need because although viruses such as SARS-CoV-2 are blocked by the filter, they will still live on the surface of the filter for a long time. This creates the possibility of secondary infection, especially when a doctor saves a patient. A recent study found that SARS-CoV-2 has extremely strong survivability in the environment [19]. SARS-CoV-2 can survive at least 3 h in aerosols, at least 72 h on the surface of plastic and steel, and even on the surface of copper with certain antibacterial properties, it can survive for more than 8 h. Therefore, in places where viruses are common, such as hospitals and patient isolation areas, anti-virus masks are needed to protect front-line medical staff and undiagnosed patients.

According to media reports, some institutions have begun to conduct corresponding research (Table 5). In addition, although not disclosed, many other institutions and companies are making efforts to manufacture anti-virus masks.

**Table 5:** Recent research on anti-virus masks reported in the media

Institution	Materials	Methods	Data availability	Media
Indian Institute of Science	Ag/Zn nanoparticle	nanofibrous polymer membrane	Issued a patent (US 9,686,997 B2); No virus test data released	Indian Institute of Science website (https://covid19.iisc.ac.in/)
Indian Institute of Technology	inorganic antiviral or antibacterial nanoparticles as well as organic antiviral and antibacterial molecules	nanofibrous polymer membrane	No virus test data released	eGov magazine website (https://egov.eletsonline.com/2020/04/dst-supports-development-of-reusable-n95-n99-mask-with-enhanced-antiviral-efficiency/)
Viroblock SA, Switzerland	A biphasic system. The first phase is composed of nonphospholipids. The second part is an aqueous phase containing a cyclodextrin derivative.	Coating	Masks comply with EN149 standard; 5 log antiviral performance; 99,999% protection from H1N1, H5N1 and human coronaviruses	T. Pelet & J. Paterson, “A Novel Technology to Protect from Airborne Viruses”, Asia–Pacific Biotech News, 2013, 17.
Hong Kong University of Science and Technology	Undisclosed	Coating	99.99% protection from bacteria and viruses including rubella, avian influenza, H1N1 and FCV	Hong Kong University of Science and Technology website
University of Kentucky	proteolytic enzymes	Antivirus filter	No virus test data released	University of Kentucky website (https://uknow.uky.edu/research/uk-researchers-seek-develop-antiviral-membrane-mask)

The efforts of institutions and companies in this area are impressive. However, there are still some issues worth noting. The current challenge in designing an anti-virus mask is that (i) it must kill the virus in a short time (ideally in a few seconds). Although there are many antiviral materials, their virus-killing speed still needs to be verified; (ii) the antiviral materials used must be safe. Media reports (Table 5) show that many institutions are using nanomaterials or nanoreagents, which have hidden dangers and require further experimental verification; (iii) the anti-viral material needs to have good durability on the mask. Related tests have not seemed to attract attention.

In addition, most current research is focused on the development or selection of antiviral materials. However, these antiviral materials may cause safety problems. With this in mind, some physical antiviral methods may be a better choice, such as using non-adhesive surfaces or manufacturing antiviral nanostructures. These physical antiviral structures will not fall off and may provide long-lasting antiviral effects. However, materials in this area still need to be developed.

In view of the important role of antivirus masks, governments and manufacturers should quickly formulate corresponding product standards, including the characterization of virus-killing speed, safety test methods and requirements for durability. In this regard, material innovation will greatly promote the development of the entire industry chain.

### Degradable masks

An increasing number of people are wearing masks to prevent the spread of the virus in the community. However, another problem caused by this approach is the accompanying “mask pollution”. Obviously, current masks have no self-cleaning function. A recent survey trip to the Soko islands in Hong Kong was conducted by an environmental group. 70 discarded masks were collected within 100 meters of the beach [20]. These masks will slowly break down into microplastics, entering food chains with devastating effects.

A degradable mask is a mask that has basic filtering performance and can be biodegraded after being discarded. From the perspective of sustainable development, degradable masks are an important part of the “masking strategy”. However, these masks involve the challenge of high manufacturing costs (from both production and materials) with the current design because this mask design requires that every part be biodegradable.

A novel mask design may solve the cost problem. Two Japanese companies are promoting the reuse of washable face masks to help address the shortage of face masks worldwide while simultaneously striving to improve the environmental footprint by using renewable biomass-based resources, including polylactic acid (PLA) [21].

**Fig. 5:**
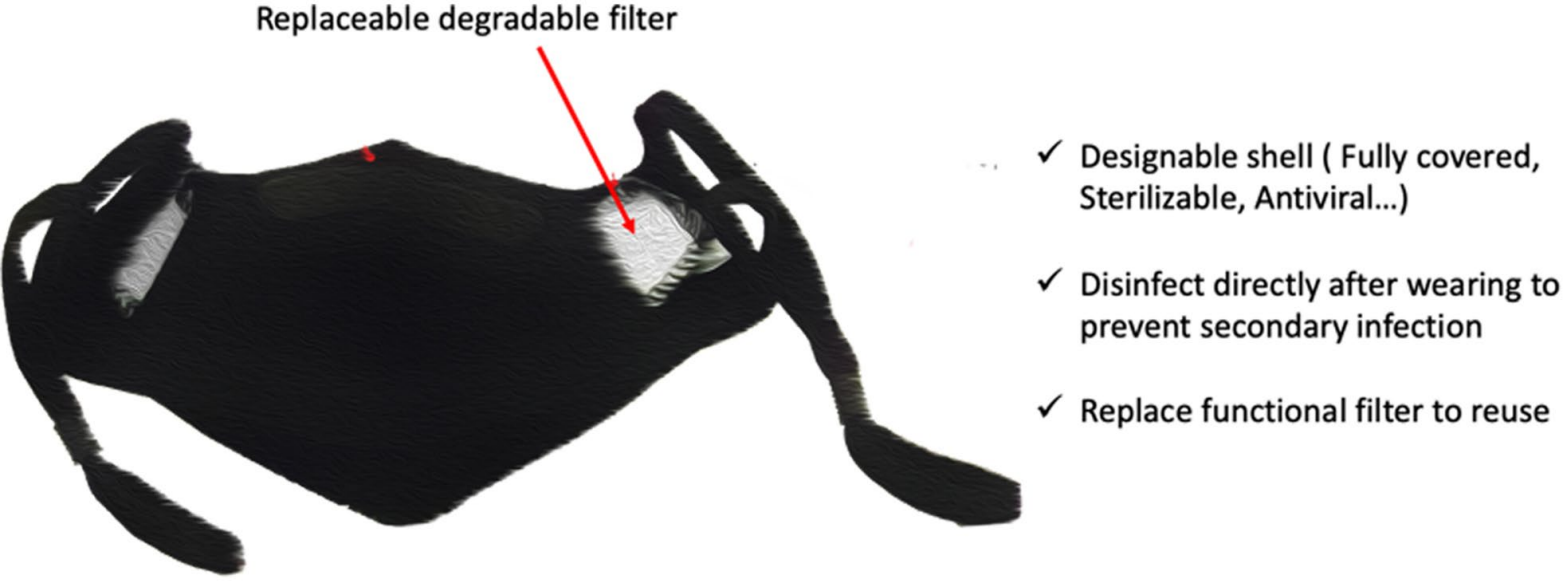
Schematic diagram of masks with replaceable filter

In addition to knitted masks, we believe that masks with replaceable filters may be a good choice (Fig. 5). The shell design of replaceable filter masks can be varied; they can fit the face better (designed separately for adults and children) and even use sterilizable materials. More importantly, they can be stored and recycled to avoid pollution caused by discarding. In this design, the filter can be prepared using degradable materials. These replaceable filter masks have the potential to reduce costs to a range acceptable to consumers while meeting environmental protection requirements. To avoid the risks of secondary infection, these masks should be disinfected after wearing. Since the filter uses degradable materials, the filter can be directly discarded after disinfection. At the same time, because there is no need to maintain the filtration efficiency of the filter, any sterilization method can be used, such as spraying medical alcohol. It is only necessary to ensure the integrity of the mask shell.

Science, business, standards, and policy innovations are needed to replace petrochemically derived plastics with degradable bioplastics derived from renewable sources [22]. Designing products with end-of-life considerations and life cycle engineering opens up opportunities for economic growth and new jobs while improving the quality of the environment.

## Conclusions

The outbreak of COVID-19 is an unprecedented challenge in recent human history. Wearing a mask has been proven to be an important means to prevent the spread of the SARS-CoV-2 virus. However, the shortage of masks is a major problem worldwide. This article provides quality information on SARS-CoV-2 virus protection with a masking strategy. We discuss possible measures to solve the shortage of masks. Disinfecting used masks using appropriate methods and making homemade masks are two emergency solutions in times of mask shortage, while innovative reusable masks, antivirus masks, and degradable masks are becoming essential for society in the long run. We also provide the definitions, basic requirements, materials, possible preparation methods and challenges of these innovative masks in this review. Science, business, standards, and policy innovations are needed to support these promising areas. Innovative masks will help us overcome the current difficulties and cope with an epidemic like COVID-19 in the future.

## Data Availability

The datasets used and/or analysed during the current study are available from the corresponding author on reasonable request.

## Abbreviations

COVID-19: pandemic coronavirus disease 2019
SARS-CoV-2: severe acute respiratory syndrome coronavirus 2
ACE2: angiotensin-converting enzyme 2
ARDS: acute respiratory distress syndrome
N95: a particulate-filtering facepiece respirator that meets the U.S. National Institute for Occupational Safety and Health (NIOSH) N95 classification of air filtration
FFP: filtering facepiece mask
AI: artificial intelligence
CDC: Centers for Disease Control and Prevention
NIOSH: U.S. National Institute for Occupational Safety and Health
VHP: Vaporous hydrogen peroxide
UVGI: ultraviolet germicidal irradiation
